# Clinicopathological comparison between PTCL‐TBX21 and PTCL‐GATA3 in Japanese patients

**DOI:** 10.1002/cam4.6793

**Published:** 2024-01-17

**Authors:** Yasumasa Shimasaki, Hiroaki Miyoshi, Keisuke Kawamoto, Noriaki Yoshida, Tatsuzo Mishina, Kazutaka Nakashima, Teppei Imamoto, Takeshi Sugio, Eriko Yanagida, Takeharu Kato, Kyohei Yamada, Mai Takeuchi, Takaharu Suzuki, Mayuko Moritsubo, Takuya Furuta, Yoshitaka Imaizumi, Jun Takizawa, Koji Kato, Junji Suzumiya, Ritsuro Suzuki, Koichi Ohshima

**Affiliations:** ^1^ Department of Pathology, School of Medicine Kurume University Kurume Japan; ^2^ Department of Hematology Shimane University Hospital Izumo Japan; ^3^ Department of Hematology, Endocrinology, and Metabolism, Faculty of Medicine Niigata University Niigata Japan; ^4^ Department of Clinical Studies Radiation Effects Research Foundation Hiroshima Japan; ^5^ Department of Hematology Chiba University Hospital Chiba Japan; ^6^ Department of Surgical Pathology Hokkaido University Hospital Sapporo Japan; ^7^ Department of Medicine and Biosystemic Science, Graduate School of Medical Sciences Kyushu University Fukuoka Japan; ^8^ Department of Hematology, Atomic Bomb Disease and Hibakusha Medicine Unit Nagasaki University Graduate School of Biomedical Sciences Nagasaki Japan; ^9^ Department of Hematology Kohga Hospital Yaizu Japan

**Keywords:** pathology, peripheral T‐cell lymphoma not otherwise specified, PTCL‐GATA3, PTCL‐TBX21

## Abstract

**Aim:**

Peripheral T‐cell lymphoma not otherwise specified (PTCL‐NOS) is a heterogeneous disease that can be classified into the PTCL‐TBX21 and PTCL‐GATA3 subtypes.

**Methods:**

In this study, we compared the clinicopathological features of PTCL‐NOS in a Japanese cohort, classified using an IHC algorithm.

**Results:**

One hundred patients with PTCL‐NOS were categorized as having PTCL‐TBX21 (*n* = 55), PTCL‐GATA3 (*n* = 24), or PTCL‐unclassified (*n* = 21). When comparing PTCL‐TBX21 and PTCL‐GATA3, PTCL‐TBX21 showed significantly lower CD4 positivity (*p* = 0.047), lower counts of high endothelial venules (*p* = 0.032), and a tendency for a better response to initial treatment (*p* = 0.088). Gene expression analysis using the nCounter system showed higher expression of tumor immunity‐related genes, such as PD‐L1, LAG3, and IDO1, in PTCL‐TBX21 than in PTCL‐GATA3. PTCL‐GATA3 had significantly worse overall survival (OS) than those with PTCL‐TBX21 (*p* = 0.047), although a similar tendency was observed for progression‐free survival (PFS) (*p* = 0.064). PTCL‐GATA3 was a prognostic factor for OS in univariate analysis (HR 2.02; 95% CI, 1.09–3.77; *p* = 0.027), although multivariate analysis did not show significance (HR 2.07; 95% CI, 0.93–4.61; *p* = 0.074). In the PFS analysis, PTCL‐GATA3 was an independent prognostic factor by univariate analysis (HR 1.96; 95% CI, 1.08–3.56; *p* = 0.027) and multivariate analysis (HR 2.34; 95% CI, 1.07–5.11; *p* = 0.032).

**Conclusion:**

The classification of PTCL‐NOS into PTCL‐TBX21 and PTCL‐GATA3 is useful for predicting the prognosis of Japanese patients and stratifying the administration of tumor immune checkpoint inhibitors in clinical practice.

## Graphical abstract

Peripheral T‐cell lymphoma (PTCL)‐TBX 21 and PTCL‐GATA3 showed clinicopathological differences including prognosis in Japanese cohort. The stratification of these subtype might be useful for clinical treatment and pathophysiological approach.

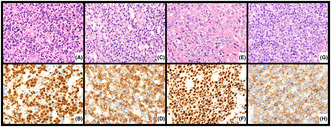

## 1 INTRODUCTION

Peripheral T‐cell lymphoma not otherwise specified (PTCL‐NOS) is defined as a mature T‐ and NK‐cell neoplasm according to the World Health Organization (WHO) classification,^1^ and is the most common subtype of PTCL, accounting for approximately 25% of all PTCLs.^2^ PTCL‐NOS is a heterogeneous disease because it is diagnosed by excluding other PTCL subtypes. The prognosis of PTCL‐NOS is usually poor, with a 5‐year overall survival (OS) of approximately 32%.^2^ Chemotherapies with cyclophosphamide, doxorubicin, vincristine, and prednisone‐like regimen showed no sufficient therapeutic effects.^2^, ^3^ Although brentuximab vedotin improved OS,^4^ more effective treatments are desired for PTCL‐NOS.

Immunological research has revealed that helper T cells (CD4^+^CD8^−^) are composed of several subtypes, including type 1 helper T cells (Th1), type 2 helper T cells (Th2), regulatory T cells (T reg), follicular helper T cells (Tfh), and type 17 helper T cells (Th17), which play different roles in the immune system. Th1‐producing interferon γ eliminates pathogen and has antitumor effects while Th2‐secreting IL4, IL5, and IL13 is involved in parasite elimination and allergy development.^5^ The transcriptional factors of Th1 and Th2 are TBX21 (T‐bet) and GATA3, respectively.^6^, ^7^ Tfh plays important roles in immunoglobulin class switching and diverse antibody production of B cells in the germinal center of the lymphoid follicle.^8^ Treg suppresses the immune responses, providing an opportunity for tumor growth.^9^ Th17 produces the inflammatory cytokines IL‐17 and IL‐22, which play an important role in pathogen elimination and autoimmune inflammation.^10^

The origin of neoplastic T cells is potentially associated with the pathophysiology of PTCLs. In the WHO classification of PTCL, angioimmunoblastic T‐cell lymphoma is categorized as PTCL of Tfh origin, together with follicular T‐cell lymphoma and nodal PTCL with TFH phenotype.^11^ Since FOXP3, a master transcriptional factor of Treg development, was expressed in patients with adult T‐cell leukemia/lymphoma (ATLL), this subtype is thought to be derived from T reg.^12^, ^13^ Previous studies revealed that PTCL‐NOS is composed of various cell origins including two biologically distinct major subtypes (PTCL‐TBX21 and PTCL‐GATA3).^14^, ^15^ Iqbal et al. reported that gene expression profiling (GEP) analysis classified PTCL‐NOS patients into two subtypes; PTCL‐TBX21 and PTCL‐GATA3, which show distinct behaviors in the prognosis and genomic alterations.^15^ The same group showed that the immunohistochemical (IHC) algorithm using T‐bet/TBX21, GATA3, CXCR3, and CCR4 also defined the two groups in patients with PTCL‐NOS.^16^ Although another study identified GATA3 expression as a marker of poor prognosis in patients with PTCL‐NOS,^17^ other clinical and pathological features of the two subtypes remain unknown.

In the present study, we compared the clinicopathological features by IHC algorithm,^16^ and tumor‐immunological gene expressions by nCounter analysis system between PTCL‐TBX21 and PTCL‐GATA3 in a Japanese cohort.

## 2 MATERIALS AND METHODS

### 2.1 Patients and samples

The study cohort comprised 100 patients newly diagnosed with PTCL‐NOS. Sixty‐seven patients were included in the International Peripheral T‐cell and Natural Killer/T‐cell Lymphoma Study,^2^ and the remaining 33 were diagnosed at the Department of Pathology, Kurume University, between 2006 and 2019. Twenty‐eight and fourteen patients were included in our previous studies.^18^, ^19^ Pathological analyses were performed using tissue microarray (TMA) samples with 2 or 3 mm core.^20^ The TMA cores were constructed by adopting representative lesion in each specimen. In analyses of morphological features, the numbers of Epstein–Barr virus (EBV)‐positive cell, plasma cell, capillary vessel, high endothelial venule (HEV), neutrophil, eosinophil, and macrophage were evaluated in high power field. The venules with enlarged nucleus and thickened eosinophilic venule wall were defined as HEV. The distinguish in EBV‐positive cells was performed by morphological findings. If EBV‐positive cells did not show neoplastic features including nuclear atypia, the cells were defined as non‐neoplastic EBV‐positive cells. All patients were reviewed by experienced hematopathologists (K.O. and H.M.) according to the WHO classification.^11^

The use of the patient sample was approved by the Research Ethics Committee of Kurume University, and the research was conducted in accordance with the guidelines of the Declaration of Helsinki. The Research Ethics Committee approved an opt‐out method for informed consent.

### 2.2 Diagnostic criteria of PTCL‐TBX21 and PTCL‐GATA3

Patients with other PTCLs, including ATLL, were excluded from this study. Patients with nodal PTCL with the TFH phenotype, expressing CD4 and three or more TFH markers, CD10, CXCL13, BCL‐6, ICOS, PD‐1, and CXCR5, were excluded.^1^

For the stratification of PTCL‐TBX21 and PTCL‐GATA3, this study adopted the IHC algorithm, as previously reported^16^ for all patients with PTCL‐NOS. Briefly, if tumor cells were more than 20% positive for T‐bet or CXCR3, they were classified as PTCL‐TBX21. In patients not classified as having PTCL‐TBX21, if tumor cells were more than 50% positive for GATA3 or CCR4, they were classified as having PTCL‐GATA3. Patients not classified as having PTCL‐TBX21 or PTCL‐GATA3 were determined as PTCL‐unclassified.^16^

### 2.3 Morphologic and IHC analysis

The antibodies used for IHC were CD3 (M7254, DAKO), CD4 (790–4423, VENTANA), CD8 (M7103, DAKO), CD30 (M0751, DAKO), TIA‐1 (IM2550, BECKMAN COULTER), Granzyme B (M7235, DAKO), T‐bet (4B10, Abcam), CXCR3 (1C6, BD), GATA3 (5852, Cell signaling Technology), and CCR4 (1G1, BD). Except for T‐bet, CXCR3, GATA3, and CCR4, the cutoff value for positivity was 30%. If neoplastic cells were positive for TIA‐1 and/or Granzyme B, the patient was considered positive for cytotoxic molecules.

### 2.4 In situ hybridization for EBV‐encoded RNA

EBV was detected using in situ hybridization with a fluorescein‐conjugated EBV peptide nucleic acid probe kit (DakoCytomatin, Glostrup, Denmark), following the manufacturer's instructions.

### 2.5 Gene expression profiling

Gene expression analysis was performed in 28 patients using the nCounter Analysis System with the PanCancer immune‐profiling panel (NanoString Technologies, Seattle, WA, USA), which consisted of 770 genes related to cancer or immune cells. Raw data from the authors' previous study were re‐analyzed by stratification between PTCL‐TBX21 and PTCL‐GATA3.^18^ Twenty‐eight patients for gene expression analysis included 21 males and 7 females with median age of 63.5 years old ranging from 7 to 80. Thirteen patients were PTCL‐TBX21, 5 patients were PTCL‐GATA3, and 10 patients were PTCL‐unclassified.

To identify genes related to PTCL‐TBX21 and PTCL‐GATA3, we compared the expression of the analyzed genes between the two groups using the Mann–Whitney *U* test. We regarded genes as characteristic of PTCL‐TBX21 or PTCL‐GATA3 when the log_2_ fold change was greater than 1.0 or less than −1.0, and the *p*‐value was less than 0.01. One‐way ANOVA was used for comparisons among the three groups.

### 2.6 Statistical analysis

Factors were compared using the chi‐square test, Fisher's exact test, and Mann–Whitney *U* test, and OS was defined as the period from the date of diagnosis to the date of the last follow‐up. Progression‐free survival (PFS) was defined as the period from diagnosis to all‐cause mortality or progression/recurrence of PTCL‐NOS. The Kaplan–Meier method was used to estimate OS and PFS, and the log‐rank test was used to compare survival curves. A Cox proportional hazards model was used to evaluate the prognostic value of each factor. Factors with significant differences in univariate analysis were adopted for multivariate analysis 1, and international prognostic (IPI) and PTCL‐GATA3 were used for multivariate analysis 2.

All *p*‐values calculated in this study were based on a two‐sided test, and *p*‐values less than 0.05 were considered statistically significant. JMP version 15.0 and R version 4.1.0 was used in all statistical analyses.

## 3 RESULTS

### 3.1 Classification of PTCL‐NOS

**FIGURE 1:**
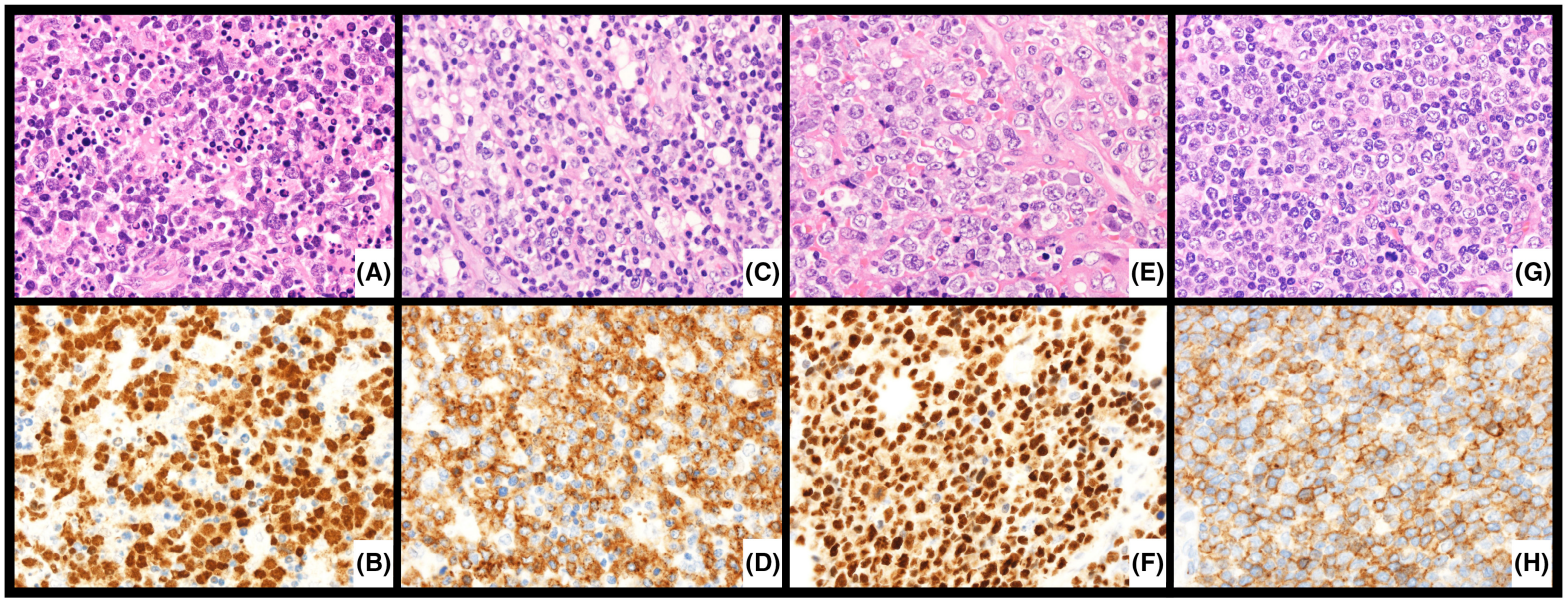
Representative hematoxylin and eosin and immunostaining for TBX21, CXCR3, GATA3, and CCR4 (Original magnification ×600). Patients with peripheral T‐cell lymphoma (PTCL)‐TBX21 showed T‐bet/TBX21 in the nucleus (A, B) and CXCR3 in the cytoplasm and/or cell membrane (C, D). Patients with PTCL‐GATA3 had GATA3 in the nucleus (E, F) and CCR4 in the cytoplasm and/or cell membrane (G, H).

According to the IHC algorithm,^16^ 55 patients were classified as PTCL‐TBX21, 24 as PTCL‐GATA3, and 21 as PTCL‐unclassified (Figure 1; Figure S1).

### 3.2 Clinical findings

PTCL‐GATA3 tended to have higher proportions of CR(u) rate at initial treatment than PTCL‐TBX21 (*p* = 0.088). There were no significant differences in other clinical findings, including age, sex, B symptoms, skin rash, splenomegaly, hepatomegaly, bone marrow involvement, peripheral blood involvement, extranodal involvement, performance status, Ann Arbor Stage, and IPI score. Blood test data include blood cell count, hemoglobin count, platelet count, elevated lactate dehydrogenase (LDH) level, hypergammaglobulinemia, and elevated CRP (Table 1).

**TABLE 1:** Statistical comparison of clinical features between peripheral T‐cell lymphoma (PTCL)‐not otherwise specified subtype.

Characteristics	PTCL‐TBX21 (%)	PTCL‐GATA3 (%)	PTCL‐unclassified (%)	*p*‐Value^a^	*p*‐Value^b^	*p*‐Value^c^
	*n* = 55	*n* = 24	*n* = 21			
Sex, male/female	31/24	14/9	12/7	0.713	0.879	0.603
Age (year), average/median [range]	64.1/69 [19–82]	66.3/68 [45–89]	58.9/59.5 [7–91]	0.930	0.053	0.049
Clinical findings						
B‐symptoms	21/35 (60.0%)	4/13 (30.8%)	4/11 (36.4%)	0.106	1.000	0.298
Skin rash	7/31 (22.6%)	4/13 (30.8%)	4/10 (40.0%)	0.706	0.685	0.413
Hepatomegaly	5/32 (15.6%)	2/13 (15.4%)	2/10 (20.0%)	1.000	1.000	1.000
Splenomegaly	5/32 (15.6%)	2/13 (15.4%)	2/10 (20.0%)	1.000	1.000	1.000
Bone marrow involvement	10/31 (32.6%)	2/12 (16.7%)	1/9 (11.1%)	0.456	1.000	0.399
Peripheral blood involvement	3/35 (8.6%)	3/13 (23.1%)	1/11 (9.1%)	0.323	0.596	1.000
Extranodal site >1	8/35 (22.9%)	2/13 (15.4%)	1/11 (9.1%)	0.706	1.000	0.421
Performance status >1	12/35 (34.3%)	2/12 (16.7%)	5/11 (45.5%)	0.302	0.193	0.722
Ann Arbor Stage III or IV	25/34 (73.5%)	9/13 (69.2%)	8/11 (72.7%)	1.000	1.000	1.000
IPI, high‐intermediate or more	19/35 (54.3%)	5/13 (38.5%)	5/11 (45.5%)	0.517	1.000	0.734
Blood examination data						
WBC, average/median [range] (×10^3^ counts/μL)	7.8/5.7 [1.2–37.18]	7.7/5.5 [0.78–36.4]	7.5/5.5 [3.1–19.6]	0.494	0.487	0.898
Hb, average/median [range] (×mg/dL)	11.4/11.7 [5.1–14.4]	11.6/11.6 [8.2–14.8]	12.2/12.8 [8.2–15.4]	0.763	0.434	0.315
Platelet, average/median [range] (×10^3^ counts/μL)	195/184 [6.8–602]	154/168 [6.5–307]	205/220 [2.9–380]	0.384	0.324	0.748
Elevated LDH level	21/35 (60.0%)	10/13 (76.9%)	2/11 (18.2%)	0.330	0.012	0.035
Hypergammaglobulinemia	9/35 (25.7%)	1/10 (10.0%)	5/8 (62.5%)	0.415	0.043	0.089
Elevated CRP level	25/34 (73.5%)	7/13 (53.8%)	7/10 (70.0%)	0.203	0.669	1.000
Treatment						
Chemotherapy	31/31 (100%)	12/13 (92.3%)	7/7 (100%)	0.296	1.000	‐
Transplantation	3/25 (12.0%)	2/9 (22.2%)	0/5 (0%)	0.591	0.506	1.000
Recurrence	15/31 (48.4%)	6/10 (60.0%)	2/7 (28.6%)	0.719	0.335	0.427
Response to initial treatment, CR or CR(u)	18/31 (58.1%)	3/12 (25.0%)	5/7 (71.4%)	0.088	0.074	0.681

Abbreviations: CR, complete response/remission; CRP, C‐reactive protein; CR(u), uncertain complete response/remission; Hb, hemoglobin; IPI, international prognostic index; LDH, lactate dehydrogenase; WBC, white blood cell.

^a^ PTCL‐TBX21 versus PTCL‐GATA3.

^b^ PTCL‐GATA3 versus PTCL‐unclassified.

^c^ PTCL‐TBX21 versus PTCL‐unclassified.

### 3.3 Pathological findings

CD4 expression was significantly lower in PTCL‐TBX21 than that in PTCL‐GATA3 (*p* = 0.047). No significant differences were observed in other pathological findings, including CD8, CD30, EBV, and cytotoxic molecules, between PTCL‐TBX21 and PTCL‐GATA3 (Table 2).

**TABLE 2:** Statistical comparison of pathological features between peripheral T‐cell lymphoma (PTCL)‐not otherwise specified subtype.

Pathology	PTCL‐TBX21(%)	PTCL‐GATA3(%)	PTCL‐unclassified (%)	*p*‐Value^a^	*p*‐Value^b^	*p*‐Value^c^
CD4 expression	39/55 (70.9%)	22/24 (91.7%)	16/21 (76.2%)	0.047	0.225	0.778
CD8 expression	19/55 (34.5%)	4/24 (16.7%)	1/21 (4.8%)	0.177	0.352	0.008
CD30 expression >30%	2/55 (3.6%)	1/24 (4.2%)	0/19 (0%)	1.000	1.000	1.000
Cytotoxic molecular expression	14/55 (25.5%)	2/24 (8.3%)	1/21 (4.8%)	0.127	1.000	0.054
Epstein–Barr virus positive	4/55 (7.3%)	0/24 (0%)	1/20 (5.0%)	0.308	0.455	1.000
Cell size, large	23/49 (46.9%)	9/20 (45.0%)	1/17 (5.9%)	0.884	0.010	0.003

^a^ PTCL‐TBX21 versus PTCL‐GATA3.

^b^ PTCL‐GATA3 versus PTCL‐unclassified.

^c^ PTCL‐TBX21 versus PTCL‐unclassified.

**FIGURE 2:**
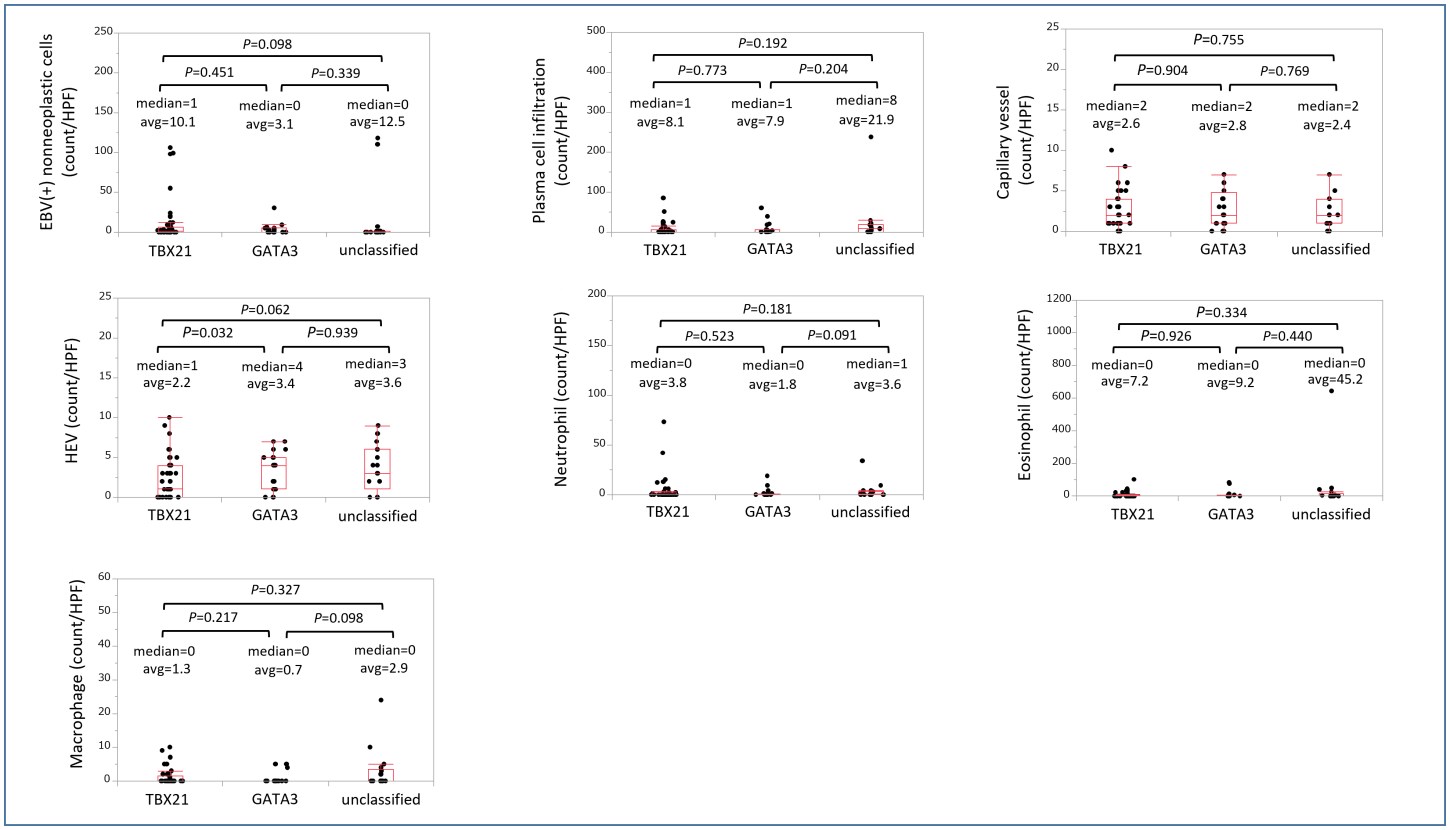
Dot plots of pathological features of tumor microenvironment. High endothelial venules (HEV) were more observed in peripheral T‐cell lymphoma (PTCL)‐TBX21 than PTCL‐GATA3 with significance (*p* = 0.032) and PTCL‐unclassified with tendency (*p* = 0.062). PTCL‐TBX21 showed a trend toward more Epstein–Barr virus (EBV)‐positive non‐neoplastic cells compared to PTCL‐unclassified (*p* = 0.098). PTCL‐unclassified showed more neutrophil (*p* = 0.091) and macrophage (*p* = 0.098) infiltration than PTCL‐GATA3.

When evaluating the tumor microenvironment, the number of HEVs was significantly lower in PTCL‐TBX21 than in GATA3 (*p* = 0.032). There were no differences in EBV‐positive non‐neoplastic cells, capillary vessels, or infiltration of plasma cells, neutrophils, eosinophils, or macrophages (Figure 2).

### 3.4 GEP of cancer immunology by nCounter system

**FIGURE 3:**
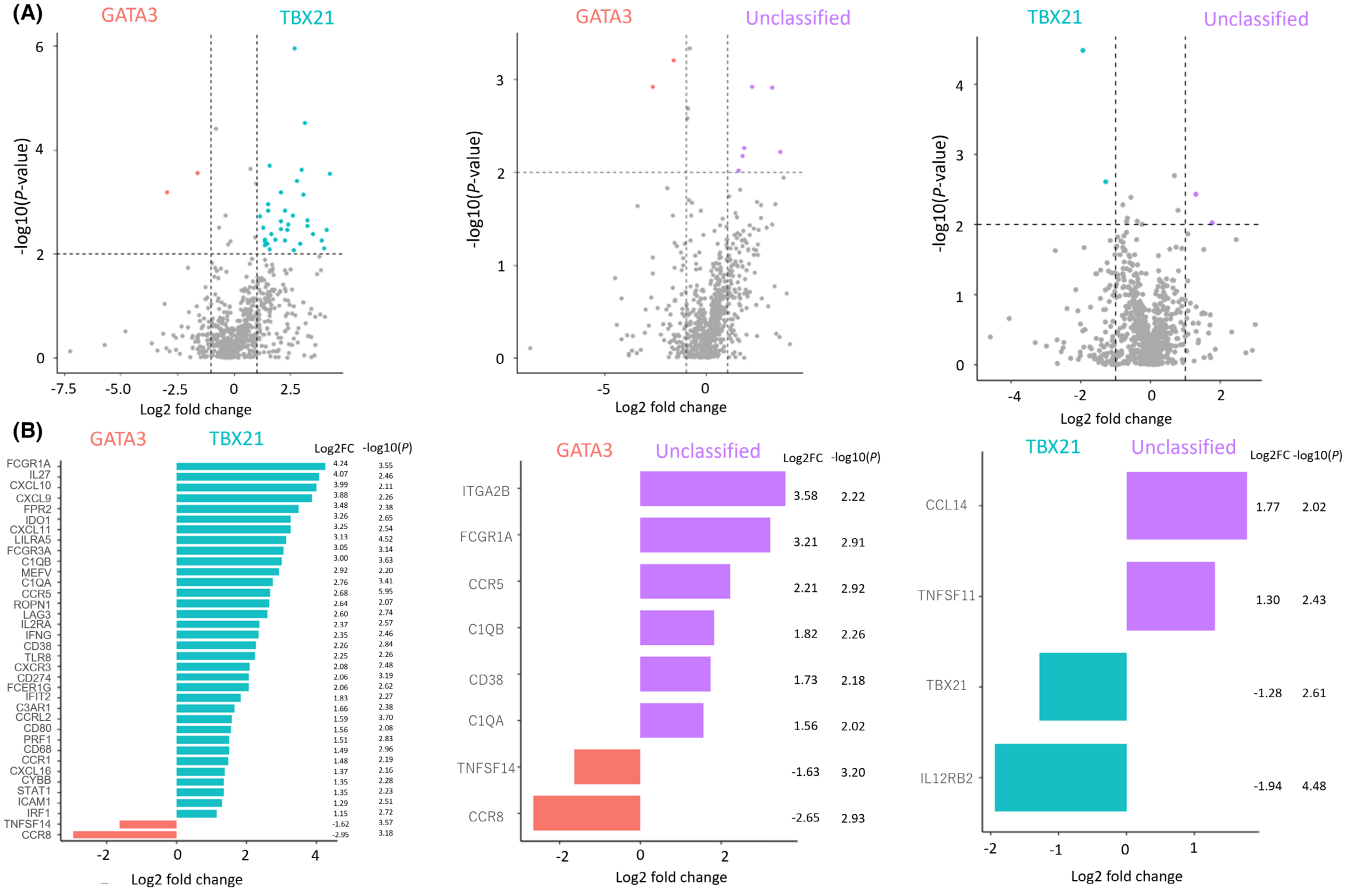
Volcano plots (A) and values of Log_2_ and fold changes of each gene (B) between peripheral T‐cell lymphoma (PTCL)‐TBX21 and PTCL‐GATA3, PTCL‐GATA3 and PTCL‐unclassified, and PTCL‐TBX21 and PTCL‐unclassified. In the analysis of PTCL‐TBX21 and PTCL‐GATA3, 34 genes were upregulated in PTCL‐TBX21 and two genes were upregulated in PTCL‐GATA3. The upregulated genes in PTCL‐TBX21 included Th1‐related genes, including CXCR3, CD38, INFG, CXCL9, CXCl11, IL27, and genes associated with tumor immunity, such as CD274 (PD‐L1), LAG3, and IDO1. In the upregulated 2 genes of PTCL‐GATA3, CCR8 is a Th2‐related gene. In the analyses between PTCL‐GATA3 and PTCL‐Unclassified and between PTCL‐TBX21 and PTCL‐Unclassified, CCR8 was upregulated in GATA3, and TBX21 was upregulated in PTCL‐TBX21.

In the volcano plot comparing PTCL‐TBX21 and PTCL‐GATA3, 34 genes were upregulated in PTCL‐TBX21, and two genes were upregulated in PTCL‐GATA3 (Figure 3A). The upregulated genes in PTCL‐TBX21 included Th1‐related genes, including CXCR3, CD38, INFG, CXCL9, CXCL11, IL27, and genes associated with tumor immunity, such as CD274 (PD‐L1), LAG3, and IDO1 (Figure 3B). In the upregulated 2 genes of PTCL‐GATA3, CCR8 is a Th2‐related gene.

**FIGURE 4:**
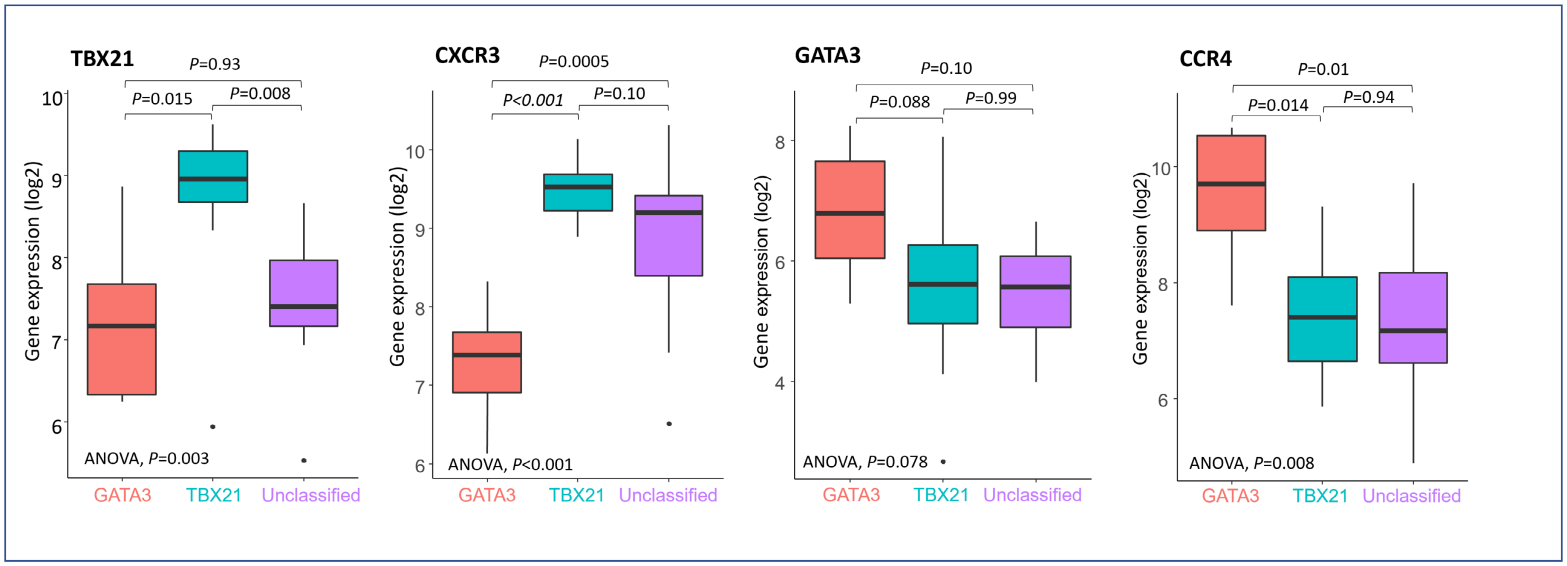
Comparisons of gene expressions of genes included in the immunohistochemical (IHC) algorithm among peripheral T‐cell lymphoma (PTCL) subtypes. TBX21 and CXCR3 were highly expressed in PTCL‐TBX21, and GATA3 and CCR4 were highly expressed in PTCL‐GATA3, consistent with the IHC algorithm.

Consistent with the IHC algorithm, TBX21 and CXCR3 were highly expressed in PTCL‐TBX21, and GATA3 and CCR4 were highly expressed in PTCL‐GATA3 (Figure 4).

### 3.5 Prognosis

**FIGURE 5:**
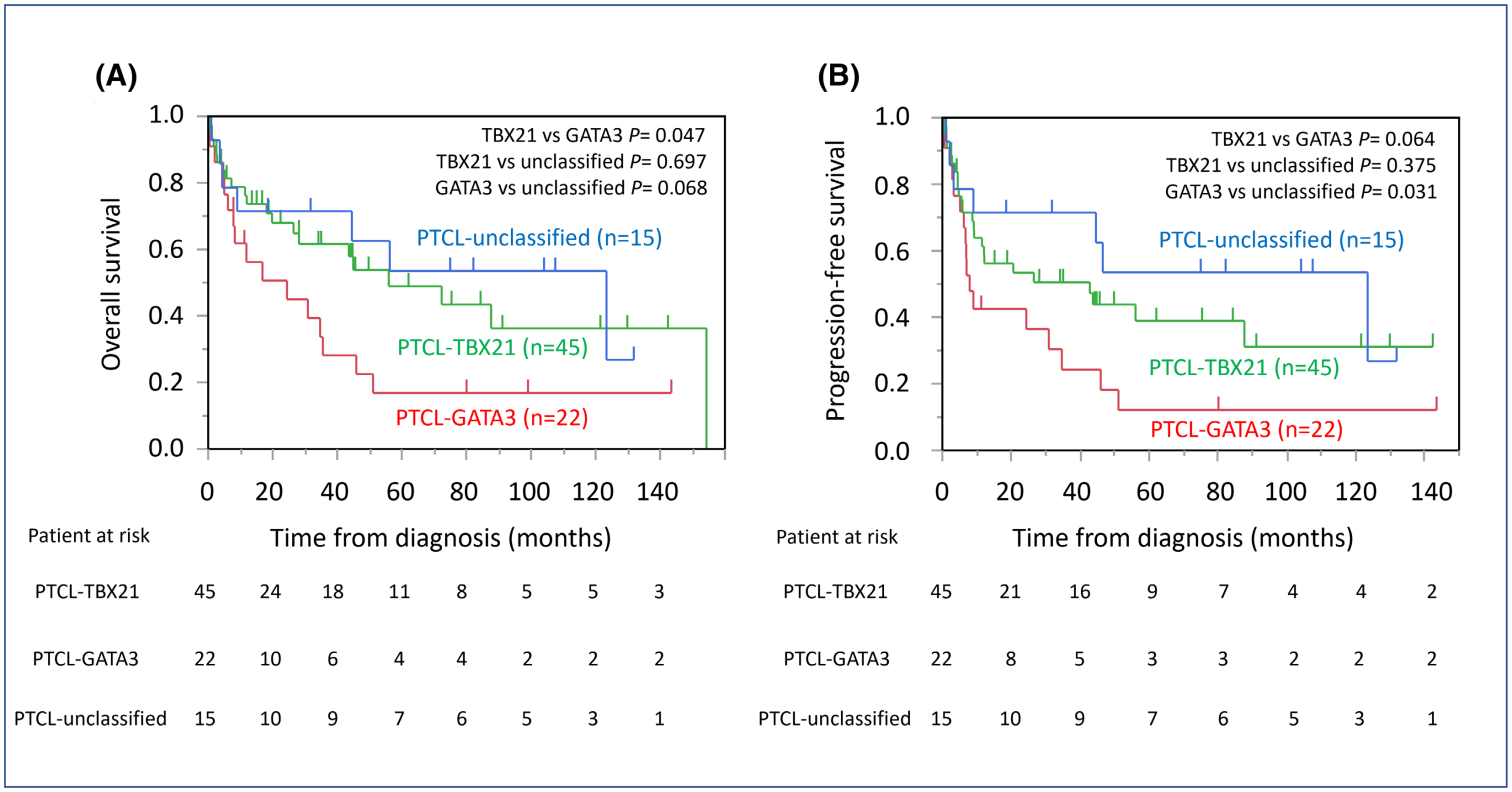
Survival curves of overall survival (OS) (A) and progression‐free survival (PFS) (B) among peripheral T‐cell lymphoma (PTCL)‐TBX21, PTCL‐GATA3, and PTCL‐unclassified. (A) PTCL‐GATA3 had significantly worse OS than PTCL‐TBX21 (*p* = 0.047), although a similar tendency was observed in PFS (*p* = 0.064). On the contrary, PTCL‐GATA3 had significantly worse PFS than PTCL‐unclassified (*p* = 0.031) although a similar tendency was observed in PFS (*p* = 0.068).

In the log‐rank test (Figure 5A,B), PTCL‐GATA3 was associated with a significantly worse OS than PTCL‐TBX21 (*p* = 0.047), although a similar tendency was observed for PFS (*p* = 0.064).

In uni‐ and multivariate analysis for OS, PTCL‐GATA3 was a prognostic factor in univariate analysis (HR 2.02; 95% CI, 1.09–3.77; *p* = 0.027), although there was no significance in multivariate analyses with other factors (Analysis 1, HR 2.09; 95% CI, 0.89–4.88; *p* = 0.090 and Analysis 2, HR 2.07; 95% CI, 0.93–4.61; *p* = 0.074) (Table 3). In the analysis of PFS, PTCL‐GATA3 was an independent prognostic factor by univariate analysis (HR 1.96; 95% CI, 1.08–3.56; *p* = 0.027) and multivariate analysis (Analysis 1, HR 2.91; 95% CI, 1.24–6.84; *p* = 0.014, and Analysis 2, HR 2.34; 95% CI, 1.07–5.11; *p* = 0.032) (Table 4).

**TABLE 3:** Univariate and multivariate analysis of overall survival of patients with peripheral T‐cell lymphoma (PTCL)‐not otherwise specified.

Variables	Unfavorable factors	Univariate analysis		Multivariate analysis 1		Multivariate analysis 2	
		HR [95% CI]	*p*‐Value	HR [95% CI]	*p*‐Value	HR [95% CI]	*p*‐Value
Age	>60 years	2.04 [1.05–3.95]	0.035	2.11 [0.94–4.79]	0.071	‐	‐
Ann Arbor Stage	III/IV	3.24 [1.12–9.33]	0.030	1.98 [0.64–6.16]	0.309	‐	‐
Performance Status	2–4	3.40 [1.60–7.25]	0.002	3.17 [1.38–7.28]	0.007	‐	‐
Extranodal disease	≧2 sites	1.89 [0.81–4.42]	0.143	‐	‐	‐	‐
Lactate dehydrogenase	>normal	0.83 [0.42–1.63]	0.586	‐	‐	‐	‐
B symptom	Present	1.65 [0.79–3.44]	0.181	‐	‐	‐	‐
BM involvement	Present	1.92 [0.88–4.16]	0.100	‐	‐	‐	‐
Cytotoxic molecule	Positive	1.93 [0.89–4.19]	0.098	‐	‐	‐	‐
IPI category	H‐I/H	1.92 [0.93–3.99]	0.079	‐	‐	2.04 [0.98–4.26]	0.058
PTCL subtype	PTCL‐GATA3	2.02 [1.09–3.77]	0.027	2.09 [0.89–4.88]	0.090	2.07 [0.93–4.61]	0.074

Abbreviations: H, high; H‐I, high‐intermediate risk; HR, hazard ratio; IPI, International Prognostic score; Others, PTCL‐TBX21 and PTCL‐unclassified.

**TABLE 4:** Univariate and multivariate analysis of progression‐free survival of patients with peripheral T‐cell lymphoma (PTCL)‐not otherwise specified.

Variables	Unfavorable factors	Univariate analysis		Multivariate analysis 1		Multivariate analysis 2	
		HR [95% CI]	*p*‐Value	HR [95% CI]	*p*‐Value	HR [95% CI]	*p*‐Value
Age	>60 years	2.24 [1.19–4.20]	0.012	2.80 [1.30–3.02]	0.009	‐	‐
Ann Arbor Stage	III/IV	4.37 [1.53–12.5]	0.006	3.58 [1.18–10.8]	0.024	‐	‐
Performance Status	2–4	2.41 [1.19–4.87]	0.015	2.00 [0.94–4.27]	0.073	‐	‐
Extranodal disease	≧2 sites	2.05 [0.92–4.54]	0.078	‐	‐	‐	‐
Lactate dehydrogenase	>normal	1.00 [0.51–1.95]	0.999	‐	‐	‐	‐
B symptom	Present	1.74 [0.88–3.43]	0.108	‐	‐	‐	‐
Bon involvement	Present	1.92 [0.93–3.98]	0.079	‐	‐	‐	‐
Cytotoxic molecule	Positive	1.70 [0.79–3.65]	0.177	‐	‐	‐	‐
IPI category	H‐I/H	1.99 [1.01–3.90]	0.045	‐	‐	2.22 [1.11–4.41]	0.023
PTCL subtype	PTCL‐GATA3	1.96 [1.08–3.56]	0.027	2.91 [1.24–6.84]	0.014	2.34 [1.07–5.11]	0.032

Abbreviations: H, high; H‐I, high‐intermediate risk; HR, hazard ratio; IPI, International Prognostic score; BM, bone marrow; Others, PTCL‐TBX21 and PTCL‐unclassified.

### 3.6 The characteristics of PTCL‐unclassified

Compared to PTCL‐TBX21, PTCL‐unclassified was significantly lower age (*p* = 0.049), had lower elevated LDH levels (*p* = 0.035), lower CD8 expression (*p* = 0.008), and smaller large cell size (*p* = 0.003). In contrast, PTCL‐unclassified had significantly lower elevated LDH levels (*p* = 0.012), higher hypergammaglobulinemia (*p* = 0.043), and smaller large cell sizes (*p* = 0.01) than PTCL‐GATA3. In the analysis of PFS, PTCL‐unclassified showed a significantly better prognosis than PTCL‐GATA3 (*p* = 0.031).

## 4 DISCUSSION

The present study classified Japanese patients with PTCL‐NOS as PTCL‐TBX21, PTCL‐GATA3, or PTCL‐unclassified using the IHC algorithm. PTCL‐TBX21 showed a good response rate to the initial chemotherapy. Patients with PTCL‐GATA3 showed higher CD4 positivity and worse PFS and OS. We confirmed that PTCL‐GATA3 was an independent poor prognostic factor for PFS.

The difference between our study and previous study of Amador et al.^16^ is that we strictly excluded TFH‐type PTCL by immunostaining for six TFH markers, including CD10, PD‐1, Bcl6, ICOS, CXCL13, and CXCR5. The previous study did not perform all of these markers for all study cohort.^16^ Although the clinicopathological differences between TFH type PTCL and PTCL‐NOS are still undetermined, this study's results are considered very meaningful because of study cohort as “true” PTCL‐NOS. On the contrary, Jain et al. has recently showed similar study.^21^ The same IHC algorithm stratified PTCL‐NOS patients into 13 patients of PTCL‐TBX21, 18 of PTCL‐GATA3, and 7 of PTCL‐unclassified.^21^ PTCL‐GATA3 had tendency of worse PFS and OS although statistical significance was not shown.^21^ The prognostic impact in this study is considered to be achieved by the larger number of patients compared with study of Jain et al. The study with many larger cohorts would confirm the result of this study in the future.

The results of protein expression by IHC algorithm^16^ were consistent with those of GEP by nCounter in this study. These results are considered to reflect the reproducibility of the IHC algorithm^16^ as alternative methods of GEP analysis. However, there might be more room for improvement because of small number of studies for validation of IHC algorithm.^16^ Further investigation could construct more appropriate IHC algorithm from clinical and pathophysiological viewpoints by evaluating cutoff value of each protein expression and adoption of more specific markers for Th1 and Th2.

Although the reason for the poor prognosis of PTCL‐GATA3 has not been fully elucidated, previous reports have investigated this point. According to a report by Tayla et al., PTCL‐GATA3 has more genetic abnormalities than PTCL‐TBX21. They also reported that mRNA of CDKN2A significantly decreased in PTCL‐GATA3.^22^ On the contrary, Watatani et al. reported that PTCL‐NOS with TP53/CDKN2A mutation has remarkable chromosomal instability and poor prognosis, although the association of *TP53/CDKN2A* mutation with PTCL‐GATA3 was not described in the Watatani study. Patients with *TP53/CDKN2A* mutation^19^ and patients with PTCL‐GATA3^22^ showed similar genomic abnormalities. Chromosomal instability associated *TP53/CDKN2A* mutation may contribute to the poor prognosis of PTCL‐GATA3.

In this study, the expression levels of tumor suppressor genes, including IDO1, CD274 (PD‐L1), and LAG3, were higher in PTCL‐TBX21. Our previous study showed that PD‐L1 and IDO1, immune checkpoint molecules, were highly expressed in tumor‐infiltrating macrophages in some patients with PTCL‐NOS.^18^ These genes are associated with mechanisms of escape from tumor immunity. PD‐L1 binds to PD‐L1 on activated T cells and inhibits antitumor immunity by counteracting T‐cell activation signals.^23^ IDO1 is an enzyme involved in tryptophan metabolism. IDO1 has been shown to be highly expressed in tumor cells of various cancers, suggesting that the depletion of tryptophan associated with increased IDO1 activity plays a vital role in suppressing tumor immunity.^24^ LAG3 is a molecule expressed on the surface of activated T cells. LAG3 inhibits T‐cell proliferation and activation, and is thought to play an important role in tumor immunity. In PTCL‐TBX21, the escape of tumor immunity may play an important role in pathogenesis.^25^, ^26^

Currently, several immune checkpoint inhibitors targeting PD‐/PD‐L, IDO1, and LAG3 have been developed, and their utility has been evaluated in several clinical trials.^27^, ^28^ Although patients with PTCL‐TBX21 showed a favorable prognosis than those with PTCL‐GATA3 in the current study. (Figure 3A), the median OS of PTCL‐TBX21 was 4.67 years from the diagnosis. Therefore, immune checkpoint inhibitors targeting these molecules may improve the prognosis of this subtype.

The present study has some limitations. First, the number of PTCL‐NOS patients was relatively small, especially for GEP analysis. Large cohort studies are warranted to confirm the results of the present study. Second, the cell of origin was classified using only the IHC algorithm. Although Amador et al. reported that the reproducibility of GEP by the IHC algorithm between PTCL‐TBX21 and PTCL‐GATA3 was approximately 85%,^16^ it is desirable to confirm the reproducibility of GEP and IHC in a Japanese cohort. Third, this study was conducted using TMA specimens. Further studies that consider lesional diversity may provide more detailed information on the pathophysiology of PTCL‐NOS.

In conclusion, the classification of PTCL‐TBX21 and PTCL‐GATA3 using the IHC algorithm for Japanese patients with PTCL‐NOS showed that they have different clinicopathological features and gene expression patterns, including tumor immune suppressor genes. These results suggest that PTCL subtyping may be useful in predicting the prognosis of Japanese patients and in stratifying the administration of tumor immune checkpoint inhibitors in clinical practice.

## AUTHOR CONTRIBUTIONS

**Yasumasa Shimasaki:** Conceptualization (equal); data curation (equal); formal analysis (equal); investigation (equal); writing – original draft (equal). **Hiroaki Miyoshi:** Conceptualization (lead); investigation (equal); project administration (equal); writing – original draft (equal); writing – review and editing (lead). **Keisuke Kawamoto:** Conceptualization (equal); data curation (equal); investigation (equal). **Noriaki Yoshida:** Data curation (equal); writing – review and editing (equal). **Tatsuzo Mishina:** Formal analysis (equal); writing – original draft (equal). **Kazutaka Nakashima:** Investigation (equal). **Teppei Imamoto:** Data curation (equal); investigation (equal). **Takeshi Sugio:** Formal analysis (equal). **Eriko Yanagida:** Data curation (equal); investigation (equal). **Takeharu Kato:** Data curation (equal). **Kyohei Yamada:** Data curation (equal). **Mai Takeuchi:** Data curation (equal). **Takaharu Suzuki:** Investigation (equal). **Mayuko Moritsubo:** Data curation (equal). **Takuya Furuta:** Data curation (equal). **Yoshitaka Imaizumi:** Data curation (equal). **Jun Takizawa:** Data curation (equal). **Koji Kato:** Data curation (equal). **Junji Suzumiya:** Data curation (equal). **Ritsuro Suzuki:** Data curation (equal). **Koichi Ohshima:** Conceptualization (equal); data curation (equal); investigation (equal).

## CONFLICT OF INTEREST STATEMENT

The authors declare no conflict of interest.

## ETHICS STATEMENT

The use of the patient sample was approved by the Research Ethics Committee of Kurume University (approval number: 439), and the Research Ethics Committee approved an opt‐out method for informed consent.

## Supporting information


Figure S1.

## Data Availability

The author elects to not share data because of privacy or ethical restrictions.
