# Supplementary material for: Clinicopathological comparison between PTCL‐TBX21 and PTCL‐GATA3 in Japanese patients
Source: Cancer Med. 2024 Jan 17;13(3):e6793. doi: 10.1002/cam4.6793 (PMC10905534; doi:10.1002/cam4.6793)
Supplement: Supplementary file 1 — Figure S1. [file CAM4-13-e6793-s001.pptx]

## Slide 1
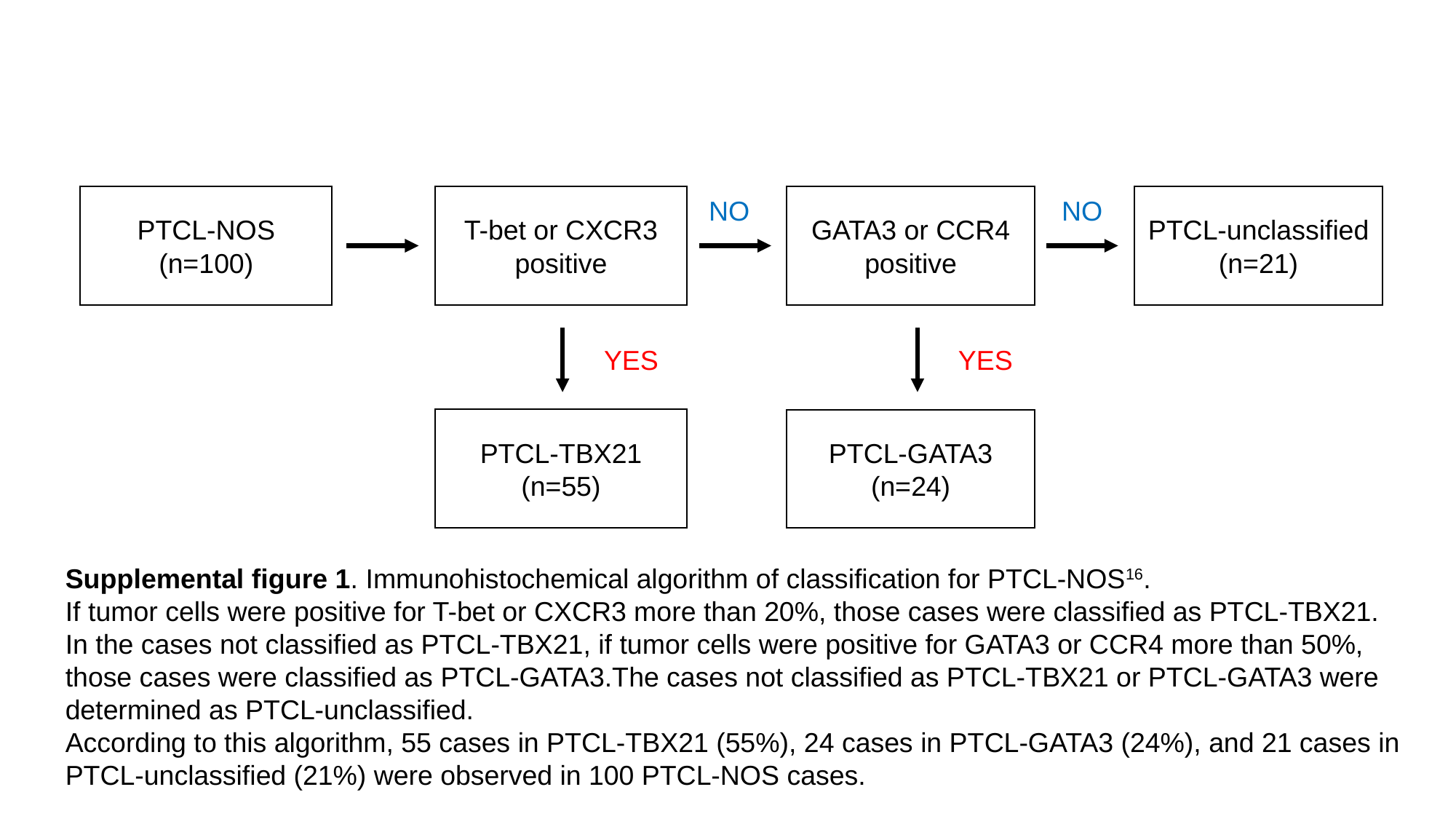

PTCL-NOS
(n=100)
T-bet or CXCR3
positive
GATA3 or CCR4
positive
PTCL-unclassified
(n=21)
NO
NO
YES
YES
PTCL-TBX21
(n=55)
PTCL-GATA3
(n=24)
Supplemental figure 1. Immunohistochemical algorithm of classification for PTCL-NOS16.
If tumor cells were positive for T-bet or CXCR3 more than 20%, those cases were classified as PTCL-TBX21.
In the cases not classified as PTCL-TBX21, if tumor cells were positive for GATA3 or CCR4 more than 50%, those cases were classified as PTCL-GATA3.The cases not classified as PTCL-TBX21 or PTCL-GATA3 were determined as PTCL-unclassified.
According to this algorithm, 55 cases in PTCL-TBX21 (55%), 24 cases in PTCL-GATA3 (24%), and 21 cases in PTCL-unclassified (21%) were observed in 100 PTCL-NOS cases.
